# Community of Practice for Nursing: connecting nurses in the Region of the Americas *

**DOI:** 10.1590/1518-8345.7254.4401

**Published:** 2024-11-15

**Authors:** Bruna Moreno Dias, Johel Nazareth Díaz Pérez, Silvia Helena de Bortoli Cassiani

**Affiliations:** ^1^ Organización Panamericana de la Salud, Departamento de Sistemas y Servicios de Salud, Washington, DC, United States of America.; ^2^ Organización Panamericana de la Salud, Panamá, Provincia de Panama, Republic of Panama.; ^3^ Organización Panamericana de la Salud, Washington, DC, United States of America.

**Keywords:** Nursing, Nursing Staff, Information Technology, Leadership, Nursing Informatics, Discussion Forums

## Abstract

to analyze the use profile and content of the messages posted in the Community of Practice for Nursing in the Region of the Americas of the Pan American Health Organization as a tool intended to share experiences, knowledge and opportunities for nurses.

a descriptive and qualitative study aimed at analyzing the messages posted in the Community of Practice forums. The data were analyzed by content analysis.

the Community of Practice has allowed the participation of 1,765 professionals from 20 countries, with a total of 4,001 posts. The topics analyzed (education, employment, leadership and service delivery) were addressed from the perspective of professionals from different countries, presenting the challenges they face and their reflections regarding the situation of nursing in the region.

the platform allowed professionals from different countries and performance scopes to interact and discuss relevant topics for nursing education and practice in the Region of the Americas. Through the Community of Practice, the expectation is to enhance commitment, cooperation and solidarity for collective learning and skills development, thus maximizing nurses’ contributions to health systems and services.

## Highlights

**(1)** The platform has allowed exchanging experiences, knowledge and opportunities.

**(2)** The participation of different actors enhances commitment and cooperation.

**(3)** It is a useful resource to discuss important topics for the nursing context.

## Introduction

 In the Region of the Americas there are around seven million nursing professionals, representing 56% of the health workforce ^(^ 1 ^)^ . These professionals are in the first line of service provision and play an important role in offering people- and community-centered care. In many countries they are leaders or key members of multidisciplinary and interprofessional health teams. Despite that, investments are still needed to strengthen and optimize this professional category ^(^ 2 ^)^ . 

Acknowledging the context marked by changes and the complexity of the decision-making process in health, in addition to proposing high-level discussions about the relevant topics for the nursing context in the Region of the Americas, the Community of Practice for Nursing in the Region of the Americas of the Pan American Health Organization (PAHO) was created.

 The Community of Practice (CoP) considers PAHO’s Strategic Directions for Nursing in the Region of the Americas ^(^ 3 ^)^ and the World Health Organization (WHO) Global Strategic Directions for Nursing and Midwifery 2021-2025 ^(^ 4 ^)^ ; it is aligned with the shared and collaborative view and need to invest in and strengthen human resources for health, specifically in nursing. 

 The CoP aims to bring together nurses and other interested parties, enhancing the exchange of experiences, information, and knowledge, and strengthening nurses’ leadership in their professional roles ^(^ 5 ^)^ . Through the community, via an online platform, it is expected to provide a space for interaction and exchange of knowledge and experiences among nurses in the Region of the Americas, in addition to collaboratively strengthening systems and services to enable universal access to health and universal health coverage and to achieve the Sustainable Development Goals. 

 The concept of Community of Practice is not new: it was first proposed in 1998 with the purpose of assembling a group of people who share common interests through dialog and collective reflection, in order to exchange knowledge and analyze proposals for practical solutions to usual problems ^(^ 6 ^)^ . 

 The objectives of a CoP can be targeted at problem-solving, brainstorming, knowledge dissemination, diffusion of lessons learned and collective reflections ^(^ 7 ^)^ . 

Thus, using Information and Communication Technology (ICT) through online forums which are easily accessible and flexible in terms of space and time, it is possible to: build support networks (networking and partnerships); learn from other experiences and propose practical solutions; critically analyze practice and maximize responses; share up-to-date materials and resources; and share professional development opportunities, such as events and training possibilities.

 Use experiences for communities of practice have been identified among teachers ^(^ 8 ^-^ 9 ^)^ , researchers ^(^ 10 ^)^ , health professionals ^(^ 11 ^-^ 12 ^)^ and nursing professionals ^(^ 13 ^)^ , although none with the scope and proposal of the CoP presented in this study. 

In view of the above, this study aims to analyze the use profile and content of the messages posted in the Community of Practice for Nursing in the Region of the Americas of the Pan American Health Organization as a tool intended to share experiences, knowledge and opportunities for nurses.

## Method

### Type of study

 A qualitative and descriptive study, carried out according to the Consolidated Criteria for Reporting Qualitative Research (COREQ) ^(^ 14 ^)^ guidelines and aimed to analyze the use profile and content of the messages posted in the Community of Practice for Nursing in the Region of the Americas of the Pan American Health Organization, created in August 2022. 

### Scenario

 The Community of Practice has been developed with the technical support of a specialist in the creation of digital learning platforms. The community is organized in a public web page ( https://comunidadenfermeria.paho.org/ ), with information about the initiative, space to announce events, additional publications and resources and connection to the PAHO Global Community of Practice for Nursing and Midwifery, in addition to the forums, available for all the participants registered in the Virtual Campus for Public Health (VCPH/PAHO). Therefore, in order to access the platform, the participants have to register as users in the VCPH, without incurring in any cost or requiring previous approval. 

 The platform offers open forums, as well as forums to introduce members and to interact in topics of interest to nursing (education, employment, leadership and service delivery), that can be accessed by all participants; it also provides restricted-access forums for specific groups that require the moderator’s approval, namely: PAHO Projects, Chief Nursing Officer and Group of Nursing Professionals from Central America and the Caribbean ( *Grupo de Profesionales de la Enfermería de Centroamérica y del Caribe* , GPECC). 

The forums are spaces that allow the participants to interact, sharing ideas, perceptions, reflections, experiences, knowledge, doubts and opportunities. In addition to messages, technical documents, articles, events and other opportunities can also be shared. In each forum, the participants can propose a new discussion or answer questions and comments already made by other peers.

**Figure 1 -:**
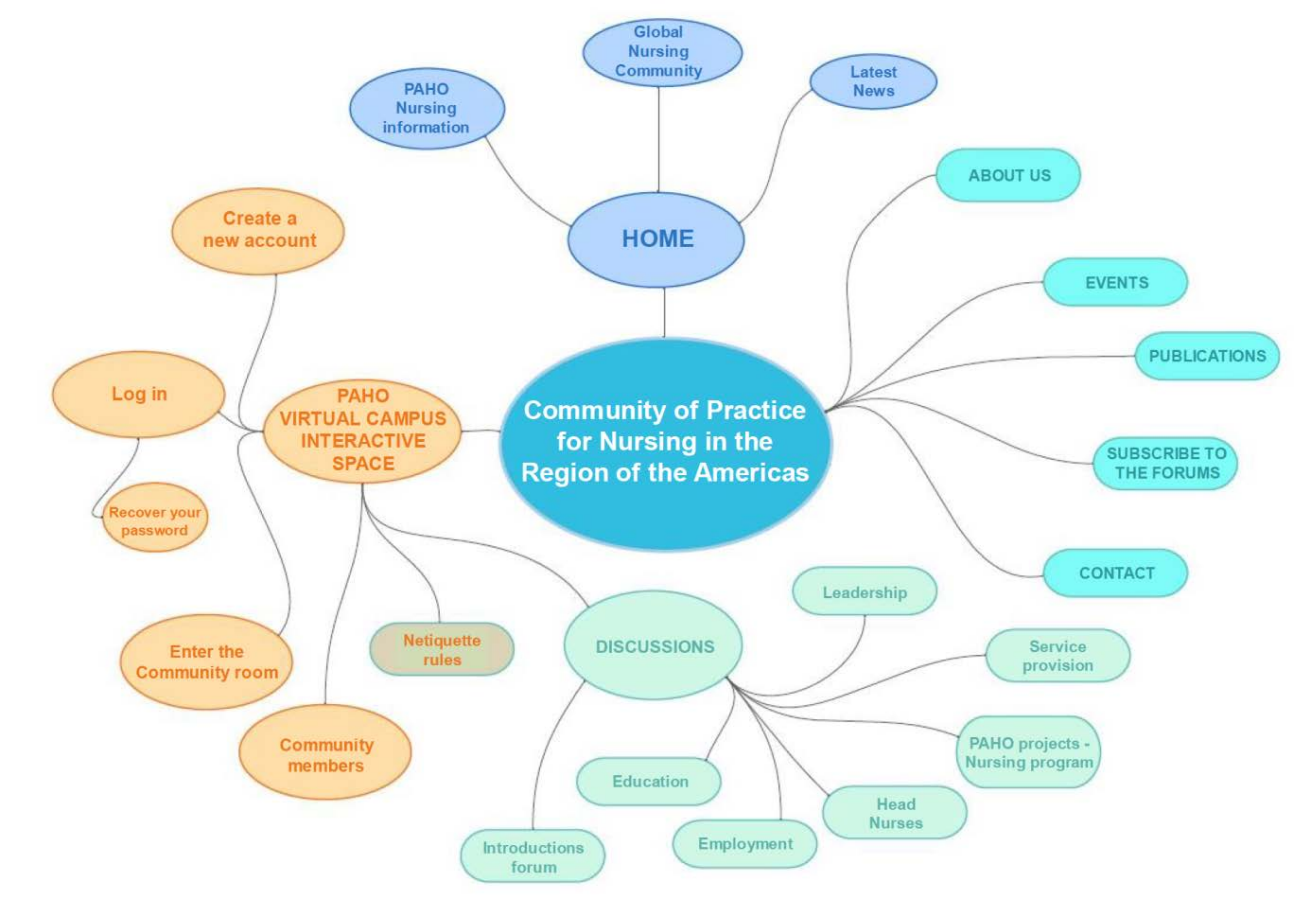
Community of Practice navigation guide. Washington, D.C., United States of America, 2023

 The CoP is also a space for training activities, allowing to take part in activities such as quizzes, webinars and reflections. The Community of Practice structure and resources are shown in Figure 1 . 

### Period and data collection

The messages analyzed were all those posted by the users (herein considered as participants of this study) between August 2022 and September 2023 in the platform’s open forums (Education, Employment, Leadership and Service delivery). The posts were extracted from the CoP platform by using the participation report tool and were organized in a text editor for their subsequent analysis.

It is worth noting that the participation report was extracted by the main researcher, who has the profile of platform administrator, using the platform’s native tool to export the forum posts to a .csv file. There was no contact between the researchers and the participants, with no possibility that the former exerted any influence on or interfered in the content. As the participants’ posts were extracted in their entirety, the reliability guarantee does not depend on their feedback or validation.

To analyze the community’s usage profile, the number of participants, country of origin, gender, age, profession, level of education and place of work were taken into account.

### Data processing and analysis

 The message records were anonymized and the participants were identified with an alphanumerical code (P1, P2, P3,...). The data were analyzed using content analysis, with lexical and semantic analysis ^(^ 15 ^)^ . The analysis categories were identified and defined beforehand ( *a priori* categories), adopting the following coding: Education, Employment, Leadership and Service delivery, for being the four strategic axes of the WHO Global Strategic Directions for Nursing and Midwifery 2021-2025 ^(^ 4 ^)^ . No subcategories were created. 

The data were analyzed by one of the researchers and validated by another. Nvivo version 14 was used to manage the data. It supports the analytical process and allows organizing data and perform a lexical, semantic and occurrence/frequency analysis of the terms.

### Ethical aspects

 The study follows the international ethical guidelines for health-related research in human beings ^(^ 16 ^)^ . For using public domain data, the study waives submission to an Ethics Committee. Nonetheless, the participants’ privacy and anonymity were preserved. It is worth noting that the community distributes contents that are sometimes provided by third parties and by its users; therefore, the opinions, pieces of advice, statements and contents expressed or made available in the Community of Practice are those of the authors themselves, not necessarily reflecting PAHO’s. 

## Results

### Use profile

 The Community of Practice consists of 1,765 participants from 20 countries in the Americas. They are mostly from Ecuador (41.8%) and Peru (30.3%), women (87.8%), aged between 26 and 35 years old (29.9%), Nursing professionals (87.1%), university graduates (57.9%) and working in hospital institutions (30.5%), as can be seen in Table 1 . 

**Table 1 -:** Characterization of the Community of Practice participants (n = 1,765). Washington, D.C., United States of America, 2023

**Variables**	**n**	**%**
**Country**		
Ecuador	737	41.8
Peru	534	30.3
Colombia	427	24.2
Mexico	13	0.7
Bolivia	13	0.7
Argentina	9	0.5
Uruguay	5	0.3
Nicaragua	4	0.2
Dominican Republic	4	0.2
Panama	2	0.1
Paraguay	2	0.1
Chile	2	0.1
Guatemala	2	0.1
Brazil	1	0.1
El Salvador	1	0.1
Cuba	1	0.1
Dominica	1	0.1
Venezuela	1	0.1
United States	1	0.1
Honduras	1	0.1
Others	4	0.2
**Gender**		
Female	1,549	87.8
Male	214	12.1
Not reported	2	0.1
**Age (years)**		
[26-35]	528	29.9
[36-45]	416	23.6
[18-25]	396	22.4
[46-55]	275	15.6
>56	143	8.1
Not reported	7	0.4
**Workplace**		
Hospital	538	30.5
Health center	470	26.6
Management and administration of health services	203	11.5
Not reported	554	31.4
**Schooling level**		
Higher Education	1,022	57.9
MSc	322	18.2
Undergraduate student	133	7.5
PhD	42	2.4
Technical Level	24	1.4
Other	206	11.7
Not reported	16	0.9
**Profession**		
Nursing professionals	1,537	87.1
Mid-level Nursing professionals	85	4.8
Generalist physicians	8	0.5
Psychologists	5	0.3
Community health workers	4	0.2
Medical interns and assistants	3	0.2
Pharmacists	3	0.2
Personal care workers in institutions	3	0.2
Traditional and alternative medicine professionals	2	0.1
Dietists and nutritionists	2	0.1
Heath and Occupational/Environmental Hygiene professionals	1	0.1
Specialized physicians	1	0.1
Social work professionals	1	0.1
Paramedical interns	1	0.1
Others	104	5.9
Not reported	5	0.3

During the period analyzed there were 4,001 messages in the CoP, of which 524 were posted in the analysis forums, namely: Education (n=163), Employment (n=104), Leadership (n=124) and Service delivery (n=133), where each forum contains the messages and opinions found in the participants’ interactions in a single communication thread.

The posts analyzed were made by 176 participants from different geographical locations, on different days and at different times and mostly in Spanish. The forums were also used to share articles and other materials.

**Figure 2 -:**
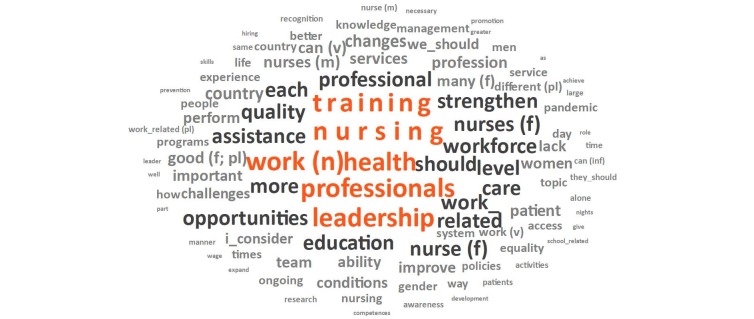
Word cloud corresponding to the Community of Practice forums. Washington, D.C., United States of America, 2023

 The mean length of the posts was 81.4 words and training, professionals, work (n), leadership, opportunities, quality and strengthen were among the most common terms, as can be seen in the word cloud presented in Figure 2 , a visual representation of the most frequent terms found in all 524 messages posted in the CoP analysis forums. 

The content analysis by topics is presented below.

### Education

As for Education, the participants highlighted the importance of a Nursing training structure that fits the health needs and a care model centered in health promotion and disease prevention; they also consider it opportune to change the current learning paradigm, focusing on the social determinants of health and on primary health care.

*Nursing programs should offer options fitted to the country’s social reality (P6).*

*Nursing education requires understanding the socioeconomic and cultural determinants of the health-disease processes; it implies a constant study of the relationship between education and work, so that the pedagogical practice drives the new way of thinking (P54).*

Special emphasis is placed on the need for continuous investments in education, with training opportunities at the different health care levels and throughout the professionals’ career.

*It is also necessary to continue with in-service education, strengthen the practice of those nurses that work in services at the different assistance levels (P18).*

To such end, although incorporating information and communication technologies is considered a challenge, it is understood that these resources are indispensable to expand the scope of the professional training and qualification actions and strategies through the virtual modality.

*It is important that, as nurses, we have the opportunity to attend virtual training sessions since, when we’re working, time doesn’t allow us to go out for in-person training opportunities (P78).*

*Information and Communication Technologies are one of the challenges and opportunities* (P120). 

In addition, it is deemed necessary to increase the number of vacancies offered and access to graduate courses, with greater investment in research and developing new knowledge and competences, as well as enabling faculty training and expanding the ability to educate new human resources in Nursing.

*Academic training is one of the challenges, especially in terms of obtaining a higher level Nursing degree, such as MSc programs; there are few and access to them is also limited* (P156). 

*There’s a long way to go in the topic of education regarding the new techniques or advances, which encourage graduate or doctoral studies, and that can be attended in the country* (P150). 

### Employment

In the topic of Employment, the intersection between the economic and social sectors is highlighted, pointing out the need for a political stance before the different political actors and decision-makers.

*Actually, the employment issue is highly complex and depends on each country’s political and social context, where we find diversity of interests among those that take part in the decision-making processes (P54).*

Gender was one of the issues that most resonated among the participants, especially the wage difference between men and women in similar job positions and the need for more women to hold leadership roles.

*It is quite evident that solving the issues that cause the compensation differences exerts an effect that goes beyond women’s pay rise: it’s one of the most important ways to fight against gender-based discrimination in the workplace and to foster gender equality* (P55). 

In addition to gender, other forms of discrimination based on situations of vulnerability were also pointed out.

*Gender inequalities are still a discussion topic and, although they persist, it should be acknowledged that some countries have been working on the issue to minimize the risks, especially in more vulnerable groups such as indigenous, afro-descendant, lesbian, gay, bisexual, transgender and intersexual (LGBTI) and migrant women; that’s why I consider that hard work is needed in educational strategies to achieve a change in the communities and in institutionality, with critical thinking to culturize and improve the social dynamics* (P22). 

The wage issue in the profession was also addressed, with discussions on the incompatibility between salaries and levels of training and experience, the recommendation to set wage floors and the negative repercussions of low remunerations.

*Earning a wage that’s in line with the training level, but this is not true many times and the professionals that invest time and money in graduate studies do so more for personal fulfillment than to achieve some type of recognition* (P80). 

Wage differences between the public and private sectors were pointed out, as well as in the different hiring modalities, with work flexibilization, underemployment, lack of occupational guarantees and low job stability. This discussion was linked to the topics of working conditions and decent work.

*There’s certain disparity in wages and hiring modalities, they even offer wages that are lower than or identical to that of a Nursing assistant* (P6). 

*The Nursing work should meet the Decent Work characteristics; this implies access to: fair wages, social security, professional training, gender equality, risk-free environments and working rights in equal conditions for all* (P133). 

### Leadership

Regarding Leadership, the participants acknowledge its importance for attaining objectives and for better performing their activities, even if this supposes a challenge for them.

*The professional practice brings new challenges with it every day, especially in reinforcing what has been learned and in relation to training, updating and preparedness to respond to the needs that may arise* (P58). 

Given the role played by nurses in health services, the need for them to have more space for leadership is highlighted, especially in primary health care facilities.

*There are no spaces for Nursing leaders in primary health care, as a starting point; therefore, they adopt this role for the sake of the profession, but with no economic or administrative recognition; instead of motivating, this can be demotivating, as leading a team implies many more responsibilities* (P147). 

*There are also no spaces for Nursing leaders in primary health care, we recognize nurses’ work in that assistance level; they’re the backbone of a Health institution, they lead the health team in the assistance provided to people, families and communities, with leadership, devotion and constancy in distant areas (sometimes even geographically inaccessible) (P21).*

To that end, nursing education needs to be improved in terms of knowledge and skills, with formal opportunities to develop leadership.

*Leadership programs should be established or organized to foster the training of young Nursing professionals so that they can become leaders* (P18). 

This includes adopting management tools, strengthening critical thinking and enhancing the ability to generate, analyze and use data for decision-making.

*Leadership training should start in the classrooms, it should strengthen critical thinking, research, innovation; we should foster expanding our knowledge* (P63). 

Finally, from the perspective of performing leadership functions, expanding nurses’ role was pointed out, by implementing the figure of Advanced Practice Nurses given their autonomous and independent performance.

*I think that strengthening the advanced practice of those Nursing professionals that care for people and populations every day would contribute to improving this leadership capability in their practical expertise field (P177).*

### Service delivery

Regarding service delivery, the health systems’ resoluteness was highlighted, especially the first level of care in network with other health services, so that nursing professionals can act adequately to meet the population health needs.

*The health systems’ response capacity implies facing several challenges such as socioeconomic and political changes, modifications in the epidemiological profile, disease outbreaks and climate change* (P3). 

*As for the Referral and counter-referral issue, there are limitations in primary care due to inadequate infrastructure and limited resoluteness of some Health institutions* (P21). 

The shortage of human resources for health, especially nursing personnel, was highlighted as a limiting factor in the provision of health services, which exerts negative impacts on the professionals’ working conditions and mental health. Likewise, difficulties related to the structure for providing assistance were addressed, with limited financial and material resources and a negative impact on safe work.

*Lack of human resources limits us to provide better quality assistance producing work overload; then Mental Health is affected, there’s a deficit in the computer systems, insufficient equipment and furniture, inadequate infrastructure* (P448). 

*The budget devoted to health should be increased since, due to lack of inputs in health institutions, it becomes difficult to provide good quality assistance* (P135). 

Finally, the regulatory aspect was addressed, highlighting the need to review the normative frameworks to update aspects such as practice environment, workload and remuneration, in addition to guaranteeing nursing workers’ rights.

*Mainly, standardizing working hours, workloads and roles, so that the Nursing work is recognized as a hub for many more professionals (P61).*

*Policies, norms, legislation, but more than all that, some compliance regulation in relation to the workload and the incentive that each professional receives, recognition of the profession and changes in the health union paradigm regarding health workers’ professional performance* (P47). 

## Discussion

The results of this analysis reinforce the usefulness of the Community of Practice as a platform to exchange information and relevant opinions for the nursing context in the Region of the Americas.

 The forums have allowed different professionals to interact, considering their local contexts, expressing themselves from different perspectives, stating their opinions and sharing experiences and suggestions. In line with the strategic directions proposed by the PAHO ^(^ 3 ^)^ and the WHO ^(^ 4 ^)^ , the topics are focused on areas that require more investments for the advancement of nursing in the Region of the Americas. 

 The CoP constitutes a safe and welcoming space for interactions, fostering participation and inclusion, promoting innovation and adaptability and reducing professional isolation, as its members develop certain sense of connection, mutual trust, reciprocity and appreciation of the different opinions, which turns it into an important tool for health professionals ^(^ 17 ^-^ 18 ^)^ . 

 Of the topics discussed, regarding education it is important to point out that the Region of the Americas presents heterogeneous training of Nursing professionals and that changes are required in the educational paradigm; these changes should take into account a model of care centered on the population health needs and emphasize strengthening the health systems with a focus on primary health care ^(^ 19 ^)^ . 

 Another issue related to education was investing in faculty training, a relevant topic, as it was observed that some of the faculty from some countries in the Region of the Americas lack any pedagogical qualification to perform their work ^(^ 20 ^)^ . 

 Thus, investing in graduate courses is a strategy to expand nurses’ training capacity, as one of the actions to face the shortage of professionals ^(^ 21 ^)^ . In addition, nurses with doctoral training contribute to improving the assistance provided to the patients and to developing health policies ^(^ 22 ^)^ . 

 Investing in this training level will also have repercussions in research development, especially in topics that contribute to universal health, so as to qualify nurses for situational analysis and for using the best evidence available to propose solutions to health problems ^(^ 23 ^)^ . 

 In this context, solving the gap in the doctoral training offer is a challenge for the Region of the Americas, as many Latin American and Caribbean countries do not offer doctoral programs and 65% of these programs are in Brazil ^(^ 2 ^)^ . 

 Doctoral training and faculty development can benefit from the Community of Practice ^(^ 24 ^)^ as an informal space for ongoing professional development, enhancing these professionals’ capabilities ^(^ 17 ^)^ , in addition to the potential of the partnerships between researchers and professionals as a way of producing knowledge that is relevant to practice ^(^ 6 ^)^ . 

 In this context, in addition to discussing problems and solutions for nursing education, the Community of Practice stands out as a dynamic and collaborative learning space where diverse knowledge and learning opportunities are shared ^(^ 13 ^)^ . 

 As for leadership, it is understood that nurses should be considered in the development and enforcement of governmental policies and that they should hold decision-making positions in the health system organizations and governmental departments; in this way, the formal strategies to encourage growth and development of the new nursing professionals are essential ^(^ 2 ^)^ . In this context, it is known that the interactions that take place in the CoP are useful for professional practice and for strengthening professional identity and role ^(^ 25 ^)^ . 

 It is important to take into account that the topics discussed in the forums are in line with the “Policy on the Health Workforce 2030: Strengthening Human Resources for Health to Achieve Resilient Health Systems”, approved in September 2023 by PAHO’s 60th Executive Council, which sets forth priority actions: a) Strengthening governance and driving nationwide policies and plans related to human resources for health; b) Developing and consolidating the regulatory mechanisms related to human resources for health; c) Strengthening the creation and integration of interprofessional teams in integrated networks of health services based on primary health care; d) Enhancing the development of health personnel capabilities and strengthening those abilities to address the populations’ priorities and for preparedness and response to public health emergencies; and e) Promoting decent work conditions and health workers’ physical and mental safeguarding, in addition to adequate staffing in human resources for health through funding and regulation ^(^ 26 ^)^ . Thus, politics presents elements and contextualizes the topics discussed in the CoP, acting as support for decision-makers and other stakeholders from different sectors to propose strategic actions targeted at the required changes in health systems, in order to strengthen human resources for health. 

Consequently, it is understood that the CoP participants prove to be aware of the practical problems related to human resources for health and nursing, exchange opinions and seek solutions to these problems. The relevance and complexity inherent to the topics treated reinforce the importance of the CoP as an interaction space, with a strong dialogical relationship.

Despite the positive results obtained after one year of having applied the CoP and of its potential uses, it is understood that commitment and sustainability of the discussions and ICT use emerge as limitations.

 Involving the participants and maintaining the discussions represent a challenge. It is understood that CoPs are “providers of answers” and that there should be questions for that; consequently, it is important to foster continuity of the discussions, feeding the group with reflections, questions and clarifications, and valuing its comments ^(^ 8 ^)^ . On the other hand, there are still frequent obstacles to using digital technologies, such as infrastructure, time, skill, training, support and familiarity with digital resources ^(^ 27 ^)^ . This requires actions targeted at digital literacy and at developing competences in terms of ICTs. Despite these challenges, it is understood that the CoP emerges as an important tool for the professionals in the Region of the Americas; thus, the expectation is to expand its scope and for more nurses to participate. 

The contribution of this study is analyzing and disseminating the use profile and the contents discussed in a Community of Practice for Nursing. From the results of this analysis, Nursing professionals can learn about and join this or other communities, researchers can adopt a similar analysis of the communication tools based on digital platforms, and decision-makers and other stakeholders can propose and incorporate ICT use in the Nursing practice, education and research scopes.

As a limitation, it is understood that the posts were analyzed without any complementary approach to explore the topics with the participants; however, it is considered that the participation of nurses from different countries and the relevance of the topics researched contribute to analyzing and reflecting on issues related to Nursing education and practice in the Region of the Americas.

## Conclusion

The objective of this study was to analyze the use profile and the content of the posts published in the PAHO Community of Practice for Nursing in the Region of the Americas.

Participants in the CoP, from 20 countries, are mostly women, aged between 26 and 35 years old, nursing professionals, university graduates and working in hospital institutions. The findings show that the online platform has allowed using easy-to access discussion forums that are flexible in terms of space, time and language, in addition to the construction of professional networks and the exchange of experiences, materials, updated resources and professional development opportunities.

The topics analyzed (education, employment, leadership and service delivery) were addressed from the perspective of professionals from different countries, presenting the challenges they face and their reflections regarding the situation of nursing in the Region. Through the Community of Practice it was possible to discuss important topics for the nursing context in the Region of the Americas and to enhance the nursing professionals’ commitment, cooperation and solidarity for collective learning and skills development, maximizing nurses’ contributions to health systems and services.

Thus, it is expected that more nurses and other stakeholders may join the Community of Practice and collaboratively contribute to the advancement of nursing in the Region of the Americas.
