# Supplementary material for: Community of Practice for Nursing: connecting nurses in the Region of the Americas
Source: Rev Lat Am Enfermagem. 2024 Nov 15;32:e4401. doi: 10.1590/1518-8345.7254.4401 (PMC11653743; doi:10.1590/1518-8345.7254.4401)
Supplement: Translation

# Translation: Comunidad de Práctica de Enfermería: conectando a las enfermeras y los enfermeros en la Región de las Américas *

**DOI:** 10.1590/1518-8345.7254.4400

**Published:** 2024-11-15

**Authors:** Bruna Moreno Dias, Johel Nazareth Díaz Pérez, Silvia Helena de Bortoli Cassiani

**Affiliations:** ^1^ Organización Panamericana de la Salud, Departamento de Sistemas y Servicios de Salud, Washington, DC, Estados Unidos de América.; ^2^ Organización Panamericana de la Salud, Panamá, Provincia de Panama, República de Panamá.; ^3^ Organización Panamericana de la Salud, Washington, DC, United States of America.

**Keywords:** Enfermería, Personal de Enfermería, Tecnología de la Información, Liderazgo, Informática Aplicada a la Enfermería, Foros de Discusión

## Abstract

analizar el perfil de uso y el contenido de los mensajes de la Comunidad de Práctica de Enfermería en la Región de las Américas de la Organización Panamericana de la Salud como herramienta destinada a compartir experiencias, conocimientos y oportunidades para las enfermeras y los enfermeros.

estudio descriptivo y cualitativo, destinado al análisis de los mensajes publicados en los foros de la Comunidad de Práctica. Los datos se analizaron mediante análisis de contenido.

la Comunidad de Práctica ha permitido la participación de 1765 profesionales de 20 países, con 4001 mensajes publicados. Los temas analizados (educación, empleo, liderazgo y prestación de servicios) se abordaron desde la perspectiva de profesionales de distintos países, presentando los retos a los que se enfrentan y sus reflexiones sobre la situación de la Enfermería en la Región.

la plataforma permitió a profesionales de diferentes países y ámbitos profesionales interactuar y debatir temas relevantes para la educación y la práctica de Enfermería en la Región de las Américas. A través de la Comunidad de Práctica se espera aumentar el compromiso, la cooperación y la solidaridad para el aprendizaje colectivo y el desarrollo de capacidades, maximizando así la contribución de las enfermeras y los enfermeros a los servicios y sistemas de salud.

## Destacados

**(1)** La plataforma ha permitido el intercambio de experiencias, conocimientos y oportunidades.

**(2)** La participación de diferentes actores aumenta el compromiso y la cooperación.

**(3)** Es un recurso útil a fin de debatir temas importantes para el contexto de la Enfermería.

## Introducción

 En la Región de las Américas hay alrededor de siete millones de profesionales de Enfermería, que representan el 56% de la fuerza de trabajo en salud ^(^ 1 ^)^ . Estos profesionales están en la primera línea de la prestación de servicios y desempeñan un papel importante en la provisión de cuidados centrados en las personas y las comunidades. En muchos países, actúan como líderes o miembros clave de equipos de salud multidisciplinarios e interprofesionales. A pesar de ello, sigue siendo necesario invertir para fortalecer y optimizar esta categoría profesional ^(^ 2 ^)^ . 

Reconociendo el contexto de cambios y la complejidad del proceso de toma de decisiones en salud y proponiendo discusiones de alto nivel sobre temas relevantes para el contexto de la Enfermería en la Región de las Américas, se desarrolló la Comunidad de Práctica de Enfermería en la Región de las Américas de la Organización Panamericana de la Salud (OPS).

 La Comunidad de Práctica (CdP) considera la Orientación estratégica para Enfermería en la Región de las Américas de la OPS ^(^ 3 ^)^ y las Orientaciones estratégicas mundiales sobre Enfermería y Partería 2021-2025 de la Organización Mundial de la Salud (OMS) ^(^ 4 ^)^ ; y se alinea con la visión compartida y colaborativa y la necesidad de invertir y fortalecer los recursos humanos para la salud, específicamente la Enfermería. 

 La CdP tiene el objetivo de reunir enfermeras y enfermeros y otros interesados, potenciar el intercambio de experiencias, información y conocimientos, y fortalecer el liderazgo de las enfermeras y los enfermeros en sus espacios profesionales ^(^ 5 ^)^ . A través de comunidad, por medio de una plataforma en línea, se espera proporcionar un espacio para la interacción y el intercambio de conocimientos y experiencias entre las enfermeras y los enfermeros en la Región de las Américas, además de fortalecer colaborativamente los sistemas y servicios para el acceso universal a la salud y la cobertura universal de salud y el logro de los Objetivos de Desarrollo Sostenible. 

 El concepto de Comunidad de Práctica no es nuevo: se propuso por primera vez en 1998 con el propósito de componer un grupo de personas que compartieran intereses comunes a través del diálogo y la reflexión colectiva, para intercambiar conocimientos y analizar propuestas de soluciones prácticas a problemas comunes ^(^ 6 ^)^ . 

 Los objetivos de una CdP pueden estar orientados a resolución de problemas, creación de ideas, distribución de conocimientos, difusión de lecciones aprendidas y reflexiones colectivas ^(^ 7 ^)^ . 

 Así, utilizando una Tecnología de la Información y la Comunicación (TIC) a través de los foros en línea, de fácil acceso y flexibles en términos de espacio y tiempo, es posible: construir redes de apoyo ( *networking* y asociaciones); aprender de otras experiencias y proponer soluciones prácticas; analizar críticamente la práctica y maximizar las respuestas; compartir materiales y recursos actualizados; y compartir oportunidades de desarrollo profesional, tales como eventos y oportunidades de capacitación. 

 Se han identificado experiencias de uso de comunidades de práctica entre docentes ^(^ 8 ^-^ 9 ^)^ , investigadores ^(^ 10 ^)^ , profesionales de la salud ^(^ 11 ^-^ 12 ^)^ y profesionales de Enfermería ^(^ 13 ^)^ , pero ninguna con el alcance y la propuesta de la CdP presentada en este estudio. 

En vista de lo expuesto, el presente estudio tiene el objetivo de analizar el perfil de uso y el contenido de los mensajes de la Comunidad de Práctica de Enfermería en la Región de las Américas de la Organización Panamericana de la Salud como herramienta destinada a compartir experiencias, conocimientos y oportunidades para enfermeras y enfermeros.

## Método

### Tipo de estudio

 Estudio cualitativo y descriptivo, realizado según las directrices *Consolidated Criteria for Reporting Qualitative Research* (COREQ) ^(^ 14 ^)^ y destinado a analizar el perfil de uso y el contenido de los mensajes la Comunidad de Práctica de Enfermería en la Región de las Américas de la Organización Panamericana de la Salud, implantada en agosto de 2022. 

### Escenario

 La Comunidad de Práctica se ha desarrollado con el apoyo técnico de un especialista en el desarrollo de plataformas digitales de aprendizaje. La comunidad se organiza en una página pública ( https://comunidadenfermeria.paho.org/ ), con información sobre la iniciativa, espacio para difundir eventos, publicaciones y recursos adicionales y conexión con la Comunidad Mundial de Prácticas de Enfermería y Partería de la OMS; además de los foros, disponibles para los participantes registrados en la plataforma del Campus Virtual de Salud Pública (CVSP/OPS). Por lo tanto, para acceder a la plataforma, el participante debe realizar el registro de usuario en el CVSP, sin incurrir en ningún coste ni necesitar aprobación previa. 

La plataforma ofrece foros abiertos, de presentación de miembros y de interacción en temas de interés para la Enfermería (educación, empleo, liderazgo y prestación de servicios), accesibles a todos los participantes, así como foros de acceso restringido, mediante aprobación del moderador, a grupos específicos: Proyectos OPS, Jefes de Enfermería y Grupo de Profesionales de la Enfermería de Centroamérica y del Caribe (GPECC).

Los foros son espacios que permiten la interacción entre los participantes, compartiendo ideas, percepciones, reflexiones, experiencias, conocimientos, dudas y oportunidades. Además de mensajes, es posible compartir documentos técnicos, artículos, eventos y otras oportunidades. En cada foro, los participantes pueden proponer un nuevo debate o responder a preguntas y comentarios formulados previamente por otros participantes.

 En la CdP también se realizan actividades de formación en este espacio, que permite participar en actividades como cuestionarios, webinarios y reflexiones. La estructura y los recursos de la Comunidad de Práctica se muestran en la Figura 1 . 

**Figura 1 -:** Guía de navegación de la Comunidad de Práctica. Washington, D.C., Estados Unidos de América, 2023

### Período y recolección de datos

Se analizaron todos los mensajes publicados entre agosto de 2022 y septiembre de 2023 en los foros abiertos de la plataforma (educación, empleo, liderazgo y prestación de servicios) realizados por los usuarios, considerados participantes de este estudio. Los mensajes se extrajeron de la plataforma de la CdP mediante la herramienta de emisión de informe de participación, y se organizaron en un editor de texto para su posterior análisis.

Cabe señalar que el informe de participación fue extraído por la investigadora principal, que tiene perfil de gestora de la plataforma, utilizando la herramienta propia de la plataforma para exportar los mensajes del foro a un archivo .csv. No hubo ningún contacto entre los investigadores y los participantes, sin posibilidad de influencia o interferencia en el contenido por parte de los investigadores. Dado que los mensajes de los participantes se extrajeron en su integridad, la garantía de fiabilidad no depende de retroalimentación ni validación de los participantes.

Para analizar el perfil de uso de la comunidad, se tuvieron en cuenta la cantidad de participantes, el país de origen, el género, la edad, la profesión, el nivel educativo y el lugar de trabajo.

### Tratamiento y análisis de datos

 Los registros de mensajes se anonimizaron y los participantes se identificaron con un código alfanumérico (P1, P2, P3, ...). Los datos se analizaron mediante análisis de contenido, con análisis léxico y semántico del contenido ^(^ 15 ^)^ . Las categorías de análisis fueron identificadas y definidas de antemano (categorías apriorísticas), adoptando los siguientes codificadores: educación, empleo, liderazgo y prestación de servicios, puesto que son los cuatro ejes estratégicos de las Orientaciones estratégicas mundiales sobre Enfermería y Partería 2021-2025 de la OMS ^(^ 4 ^)^ . No se crearon subcategorías. 

Los datos fueron analizados por una investigadora y validados por otra. Para manejar los datos se utilizó el programa informático Nvivo, versión 14, que apoya el proceso analítico y permite organizar los datos y realizar el análisis léxico, semántico y de ocurrencia/frecuencia de términos.

### Aspectos éticos

 El estudio sigue las pautas éticas internacionales para la investigación relacionada con la salud en seres humanos ^(^ 16 ^)^ . Debido al uso de datos de acceso público, el estudio está exento de presentación a un Comité de Ética. No obstante, se preservó la privacidad y el anonimato de los participantes. Cabe señalar que la comunidad distribuye contenidos a veces proporcionados por terceros y por sus usuarios; por lo tanto, las opiniones, consejos, declaraciones y contenidos expresados o puestos a disposición en la Comunidad de Práctica son los de los autores y no reflejan necesariamente los de la OPS. 

## Resultados

### Perfil de uso

 La Comunidad de Práctica está formada por 1765 participantes de 20 países de las Américas. En su mayoría son de Ecuador (41,8%) y Perú (30,3%), mujeres (87,8%), con edades comprendidas entre 26 y 35 años (29,9%), profesionales de Enfermería (87,1%), con titulación universitaria (57,9%) y que trabajan en instituciones hospitalarias (30,5%), como se observa en la Tabla 1 . 

**Tabla 1 -:** Caracterización de los participantes de la Comunidad de Práctica (n = 1765). Washington, D.C., Estados Unidos de América, 2023

**Variables**	**n**	**%**
**País**		
Ecuador	737	41,8
Perú	534	30,3
Colombia	427	24,2
México	13	0,7
Bolivia	13	0,7
Argentina	9	0,5
Uruguay	5	0,3
Nicaragua	4	0,2
República Dominicana	4	0,2
Panamá	2	0,1
Paraguay	2	0,1
Chile	2	0,1
Guatemala	2	0,1
Brasil	1	0,1
El Salvador	1	0,1
Cuba	1	0,1
Dominica	1	0,1
Venezuela	1	0,1
Estados Unidos	1	0,1
Honduras	1	0,1
Otros	4	0,2
**Género**		
Femenino	1549	87,8
Masculino	214	12,1
No informado	2	0,1
**Edad (años)**		
[26-35]	528	29,9
[36-45]	416	23,6
[18-25]	396	22,4
[46-55]	275	15,6
>56	143	8,1
No informado	7	0,4
**Lugar donde labora**		
Hospital	538	30,5
Centro de salud	470	26,6
Gestión y administración de los servicios de salud	203	11,5
No informado	554	31,4
**Nivel educativo**		
Universitario	1022	57,9
Maestría	322	18,2
Estudiante de Pregrado	133	7,5
Doctorado	42	2,4
Técnico	24	1,4
Otro	206	11,7
No informado	16	0,9
**Profesión**		
Profesionales de Enfermería	1537	87,1
Profesionales de nivel medio de Enfermería	85	4,8
Médicos generales	8	0,5
Psicólogos	5	0,3
Trabajadores comunitarios de la salud	4	0,2
Practicantes y asistentes médicos	3	0,2
Farmacéuticos	3	0,2
Trabajadores de los cuidados personales en instituciones	3	0,2
Profesionales de medicina tradicional y alternativa	2	0,1
Dietistas y nutricionistas	2	0,1
Profesionales de la salud y la higiene laboral y ambiental	1	0,1
Médicos especialistas	1	0,1
Profesionales del trabajo social	1	0,1
Practicantes paramédicos	1	0,1
Otros	104	5,9
No informado	5	0,3

Durante el periodo analizado se publicaron 4001 mensajes en la CdP, de los cuales 524 se hicieron en los foros en análisis, a saber: educación (n=163), empleo (n=104), liderazgo (n=124) y prestación de servicios (n=133), donde cada foro contiene los mensajes y las opiniones en la interacción de los participantes en un hilo de comunicación.

Los mensajes en análisis fueron realizados por 176 participantes de distintas ubicaciones geográficas, en días y horas distintos y mayoritariamente en español. Los foros también se utilizaron para compartir artículos y otros materiales.

 Los mensajes tuvieron una extensión media de 81,4 palabras y, entre los términos más comunes estuvieron formación, profesionales, trabajo, liderazgo, oportunidades, calidad y fortalecer, como puede verse en la nube de palabras de la Figura 2 , que representa visualmente los términos más comunes que se observaron en los 524 mensajes publicados en los foros en análisis de la CdP. 

**Figura 2 -:** Nube de palabras de los foros de la Comunidad de Práctica. Washington, D.C., Estados Unidos de América, 2023

A continuación se presenta el análisis de contenido por temas.

### Educación

En cuanto a la educación, los participantes destacaron la importancia de que la formación de Enfermería se ajuste a las necesidades de salud y a un modelo de cuidados centrado en la promoción de la salud y la prevención de enfermedades; también consideran oportuno cambiar el paradigma docente actual, centrándose en los determinantes sociales de la salud y la atención primaria.

*Se necesita que los programas de Enfermería oferten opciones que se ajusten a la realidad social del país* (P6). 

*La educación de Enfermería requiere comprensión en los determinantes socioeconómicos y culturales de los procesos de salud-enfermedad, implica el estudio constante de la relación entre la educación y el trabajo, a fin de que la práctica pedagógica impulse la nueva forma de pensar* (P54). 

Se hace hincapié en la necesidad de invertir continuamente en educación, con oportunidades de formación en los distintos niveles de la atención a la salud y a lo largo de toda la vida profesional.

*También es necesario continuar con la educación en servicio, fortalecer la práctica de las enfermeras que se encuentran en los servicios de los diferentes niveles de atención* (P18). 

Para ello, aunque se la incorporación de las tecnologías de la información y la comunicación considera un reto, se entiende que estos recursos son imprescindibles para ampliar el alcance de las acciones y estrategias de formación y capacitación profesional a través de la virtualidad.

*Es importante que las enfermeras y enfermeros podamos tener oportunidad de capacitaciones virtuales, ya que el tiempo cuando estamos laborando no nos permite salir a capacitarnos de forma presencial* (P78). 

*Uno de los retos y oportunidades son las tecnologías para la información y comunicación* (P120). 

Además, se presenta como necesario aumentar la oferta de plazas y el acceso a los cursos de posgrado, con mayor inversión en investigación, desarrollando nuevos conocimientos y competencias, así como posibilitando la formación del profesorado y ampliando la capacidad de formar nuevos recursos humanos en Enfermería.

*Uno de los retos es la formación académica, sobre todo en cuanto a la obtención de un título de mayor nivel en Enfermería como las maestrías, existen pocos y su acceso también es limitado* (P156). 

*Falta mucho en el tema de educación de las nuevas técnicas o los avances, que incentiven a un posgrado o doctorado, y que se puedan realizar dentro del país* (P150). 

### Empleo

En el tema del empleo, se refuerza la intersección de los sectores económico y social, señalando la necesidad de un posicionamiento político ante los diferentes actores y responsables políticos.

*Más la temática del empleo es de gran complejidad y depende del contexto político y social de cada país, donde hay diversidad de intereses entre quienes participan de la toma de decisiones* (P54). 

El género fue uno de los puntos que más resonó entre los participantes, especialmente la diferencia salarial entre mujeres y hombres en puestos similares y la necesidad de que más mujeres ocupen puestos de liderazgo.

*Es muy evidente que resolver las cuestiones causantes de las diferencias de remuneración tiene una repercusión que va más allá del aumento salarial de las mujeres: es una de las formas más importantes de luchar contra la discriminación basada en el sexo en el lugar de trabajo y de fomentar la igualdad de género* (P55). 

Además del género, también se señalaron otras formas de discriminación basadas en situaciones de vulnerabilidad.

*Las desigualdades de género siguen siendo tema de discusión y, a pesar de que persisten, se debe reconocer que en algunos países se viene trabajando en el tema para minimizar los riesgos especialmente en grupos más vulnerables como mujeres indígenas, afrodescendientes, lesbianas, gays, bisexuales, transgénero e intersexuales (LGBTI) y migrantes, por lo que considero se debe trabajar fuertemente en estrategias educativas para el cambio en las comunidades y la institucionalidad con pensamiento crítico para culturizar y mejorar las dinámicas sociales* (P22). 

También se abordó la problemática salarial de la profesión, con debates sobre la incompatibilidad entre salarios y niveles de formación y experiencia, la recomendación de establecer pisos salariales y las repercusiones negativas de los salarios bajos.

*A contar con un salario que se corresponda con el nivel de formación, pero muchas veces esto no se cumple y los profesionales que invierten tiempo y dinero en posgrados, lo hacen más por satisfacción personal que por lograr algún tipo de reconocimiento* (P80). 

Se señalaron diferencias salariales entre el sector público y el privado, así como en los distintos modelos de contratación, con flexibilización laboral, subempleo, falta de garantías laborales y poca estabilidad en el empleo. Este debate estuvo vinculado al tema de las condiciones laborales y el trabajo decente.

*Existe cierta disparidad en los salarios y en el tipo de contratación, inclusive llegan a ofrecerles salarios más bajos o iguales que a una auxiliar de Enfermería* (P6). 

*El trabajo de Enfermería debe de cumplir con las características del Trabajo Decente, lo que implica el acceso a: salarios justos, seguridad social, formación profesional, igualdad de género, libre de riesgos y derechos laborales en igualdad de circunstancias para todas y todos* (P133). 

### Liderazgo

En cuanto al liderazgo, los participantes reconocen su importancia para la consecución de objetivos y el mejor desempeño de sus actividades, aunque les suponga un reto.

*El ejercicio profesional trae consigo nuevos retos a diario, especialmente en afianzamiento de lo aprendido y de formación, actualización y preparación para dar respuesta a las necesidades que se presenten* (P58). 

Dado el papel que desempeñan las enfermeras y los enfermeros en los servicios de salud, se destaca la necesidad de que tengan más espacio para el liderazgo, sobre todo en los servicios de primer nivel.

*No existen espacios para las líderes de Enfermería en el primer nivel de atención de la salud, como una partida, por lo que asumen este rol lo hacen por la profesión, pero sin reconocimiento económico ni administrativo, en lugar de motivar puede desmotivar, ya que llevar un equipo implica muchas más responsabilidades* (P147). 

*Tampoco existen espacios de líderes en Enfermería en el primer nivel de atención de la salud, reconocemos el trabajo que realiza la enfermera en este nivel de atención; es ella la columna vertebral de un Establecimiento de salud, de llevar al frente al equipo de salud en la atención al individuo, la familia y la comunidad con liderazgo, entrega, constancia en las zonas alejadas y hasta a veces inaccesibles geográficamente* (P21). 

Para ello es necesario mejorar la capacitación del personal de Enfermería, en términos de conocimiento y habilidades, con oportunidades formales de desarrollo del liderazgo.

*Deberían establecerse u organizarse programas de liderazgo para fomentar la formación de líderes entre los profesionales jóvenes de Enfermería* (P18). 

Esto incluye adoptar herramientas de gestión, reforzar el pensamiento crítico y aumentar la capacidad de generar, analizar y utilizar datos para la toma de decisiones.

*El liderazgo debe iniciar su formación desde las aulas, se debe fortalecer el pensamiento crítico, la investigación, la innovación, debemos fomentar el extender nuestros conocimientos* (P63). 

Por último, desde la perspectiva del desempeño del liderazgo, se señaló la ampliación del rol de las enfermeras y los enfermeros, mediante la implantación del rol de Enfermera(o) de Práctica Avanzada, en vista de su actuación autónoma e independiente.

*Considero que fortalecer la práctica avanzada de los profesionales de Enfermería que están día a día con los sujetos y las poblaciones de cuidado contribuiría a mejorar esa capacidad de liderazgo en su campo de experticia práctica* (P177). 

### Prestación de servicios

En cuanto a la prestación de servicios, se destacó la capacidad resolutiva de los sistemas de salud, en especial del primer nivel de atención en red con los demás servicios de salud, a fin de que los profesionales de Enfermería puedan actuar adecuadamente para atender a las necesidades de salud de la población.

*La capacidad de respuesta de los sistemas de salud implica enfrentar varios desafíos, como cambios socioeconómicos y políticos, modificaciones en el perfil epidemiológico, brotes de enfermedades y cambio climático* (P3). 

*En el tema de Referencia y contra referencia se tienen limitaciones en el primer nivel de atención de la salud por la inadecuada infraestructura y capacidad resolutiva limitada de algunos Establecimientos de Salud* (P21). 

La escasez de recursos humanos para la salud, especialmente de personal de Enfermería, se destacó como un factor limitante en la prestación de servicios de salud, lo que genera impactos negativos en las condiciones laborales y en la salud mental de los profesionales. Del mismo modo, se abordaron las dificultades relacionadas con la estructura para prestar asistencia, con recursos financieros y materiales limitados, y un impacto negativo en el trabajo seguro.

*La falta de recursos humanos nos limita a brindar una mejor calidad de atención produciendo una sobrecarga laboral, por lo cual la Salud Mental se ve afectada, hay un déficit en los sistemas informáticos, carencia de equipos y mobiliarios, infraestructura inadecuada* (P448). 

*Se debe incrementar el presupuesto destinado a la salud pues, debido a la falta de insumos en los establecimientos de salud, se dificulta poder brindar atención de calidad* (P135). 

Por último, se abordó la regulación, destacando la necesidad de revisar los marcos normativos para actualizar aspectos como el ámbito de la práctica, la carga de trabajo y la remuneración, además de garantizar los derechos de los trabajadores de Enfermería.

*Principalmente estandarizar horas de trabajo, cargas laborales y roles, para que así se reconozca el trabajo de la Enfermería como un centro de muchos más profesionales* (P61). 

*Políticas, normas, legislación, pero más que todo eso una regulación en el cumplimiento en relación a la carga laboral y el incentivo que recibe cada profesional, el reconocimiento de la profesión y cambios de paradigma del gremio de salud en relación al quehacer profesional de los trabajadores del área* (P47). 

## Discusión

Los resultados de este análisis refuerzan la utilidad de la Comunidad de Práctica como plataforma de intercambio de información y opiniones relevantes para el contexto de la Enfermería en la Región de las Américas.

 Los foros han permitido que interactúen diferentes profesionales, considerando sus contextos locales, expresándose desde diferentes perspectivas, manifestando sus opiniones y compartiendo experiencias y sugerencias. Alineados con las orientaciones estratégicas de la OPS ^(^ 3 ^)^ y de la OMS ^(^ 4 ^)^ , los temas se centran en áreas que requieren más inversión para el avance de la Enfermería en la Región de las Américas. 

 La CdP constituye un espacio seguro y acogedor para las interacciones, fomentando la participación y la inclusión, promoviendo la innovación y la adaptabilidad y reduciendo el aislamiento profesional, ya que los miembros desarrollan cierto sentido de conexión, confianza mutua, reciprocidad y valoración de las distintas opiniones, lo que lo convierte en una importante herramienta para los profesionales de la salud ^(^ 17 ^-^ 18 ^)^ . 

 De los temas debatidos, en cuando a la educación es importante señalar que la Región tiene una formación heterogénea de profesionales de Enfermería, y que se necesitan cambios en el paradigma educativo que consideren el modelo de atención centrado en las necesidades de salud de la población y enfaticen el fortalecimiento de los sistemas de salud con un enfoque en la atención primaria ^(^ 19 ^)^ . 

 Otro tema relacionado con la educación fue la inversión en la formación del profesorado, un tópico relevante dado que se ha observado que parte de los profesores de países de la Región no han recibido cualificación pedagógica alguna para su trabajo ^(^ 20 ^)^ . 

 Así, invertir en cursos de posgrado es una estrategia para ampliar la capacidad formativa de las enfermeras y los enfermeros, como una de las acciones para hacer frente a la escasez de profesionales ^(^ 21 ^)^ . Además, las enfermeras y los enfermeros con formación doctoral contribuyen a mejorar la atención al paciente y a elaborar políticas sanitarias ^(^ 22 ^)^ . 

 Invertir en este nivel de formación también repercutirá en el desarrollo de la investigación, especialmente en temas que contribuyen a la salud universal, con el fin de capacitar a enfermeras y enfermeros para el análisis situacional y el uso de las mejores evidencias a fin de proponer soluciones a los problemas que afrontan los servicios de salud ^(^ 23 ^)^ . 

 En este contexto, resolver la brecha en la oferta de formación doctoral es un reto para la Región, ya que muchos países de América Latina y el Caribe no ofrecen programas de doctorado, y el 65% de los programas están en Brasil ^(^ 2 ^)^ . 

 La formación doctoral y el desarrollo del profesorado pueden beneficiarse de la Comunidad de Práctica ^(^ 24 ^)^ como espacio informal de desarrollo profesional continuo, aumentando la capacidad de estos profesionales ^(^ 17 ^)^ , además del potencial de las asociaciones entre investigadores y profesionales como forma de producir conocimientos pertinentes para la práctica ^(^ 6 ^)^ . 

 En este contexto, además de debatir problemas y soluciones para la formación de Enfermería, la Comunidad de Práctica se destaca como un espacio de aprendizaje dinámico y colaborativo en el que se comparten conocimientos y oportunidades de aprendizaje ^(^ 13 ^)^ . 

 En cuanto al liderazgo, se entiende que las enfermeras y los enfermeros deben ser considerados en el desarrollo y la aplicación de las políticas gubernamentales y que deben estar presentes en puestos decisorios en las organizaciones del sistema de salud y departamentos gubernamentales; de esta manera, las estrategias formales para estimular el crecimiento y el desarrollo de las nuevos profesionales de Enfermería son esenciales ^(^ 2 ^)^ . En este contexto, se sabe que las interacciones que se llevan a cabo en la CdP son útiles para la práctica profesional y para reforzar la identidad y la función profesionales ^(^ 25 ^)^ . 

 Es importante tener presente que los temas tratados en los foros están alineados con la “Política sobre el personal de salud 2030: fortalecimiento de los recursos humanos para la salud a fin de lograr sistemas de salud resilientes”, aprobada por el 60º Consejo Directivo de la OPS en septiembre de 2023, que plantea acciones prioritarias: a) Fortalecer la gobernanza e impulsar políticas y planes nacionales de recursos humanos para la salud; b) Desarrollar y consolidar los mecanismos regulatorios relacionados con recursos humanos para la salud; c) Fortalecer la conformación e integración de equipos interprofesionales en redes integradas de servicios de salud basados en atención primaria de la salud; d) Potenciar el desarrollo y fortalecer las capacidades del personal de salud para abordar las prioridades de las poblaciones y para la preparación y respuesta ante emergencias de salud pública; y e) Promover condiciones de trabajo decente y la protección física y mental de los trabajadores de salud, además de una dotación adecuada de recursos humanos para la salud mediante financiamiento y regulación ^(^ 26 ^)^ . Así, la política presenta elementos y contextualiza los temas debatidos en la CdP, sirviendo de subsidio para que los responsables de tomar decisiones y otras partes interesadas de diversos sectores propongan acciones estratégicas dirigidas a los cambios necesarios en los sistemas de salud a fin de fortalecer los recursos humanos para la salud. 

De este modo, se entiende que los participantes de la CdP demuestran ser conscientes de los problemas prácticos de los recursos humanos para la salud y la Enfermería, intercambian opiniones y buscan soluciones a estos problemas. La relevancia y complejidad de los temas tratados refuerzan la importancia de la CdP como espacio de interacción, con una fuerte relación dialógica.

A pesar de los resultados positivos obtenidos en un año de aplicación de la CdP y de sus usos potenciales, se entiende que el compromiso y la sostenibilidad de los debates y el uso de las TIC se consolidan como limitaciones.

 Involucrar a los participantes y mantener los debates es un reto. Se entiende que las CdP son “proveedoras de respuestas” y, para ello, debe haber preguntas; en consecuencia, es importante fomentar la continuidad de los debates, alimentando al grupo con reflexiones, preguntas y aclaraciones y valorando sus comentarios ^(^ 8 ^)^ . Por otra parte, siguen existiendo obstáculos frecuentes para el uso de las tecnologías digitales, entre los que se incluyen la infraestructura, el tiempo, la habilidad, la formación, el apoyo y la familiaridad con los recursos digitales ^(^ 27 ^)^ . Eso requiere acciones dirigidas a la alfabetización digital y al desarrollo de las competencias en TIC. A pesar de estos retos, se entiende que la CdP se constituye como una importante herramienta para los profesionales de la Región; así, se espera ampliar el alcance y la participación de más enfermeras y enfermeros. 

La contribución de este estudio es analizar y difundir el perfil de uso y los contenidos debatidos en una Comunidad de Práctica de Enfermería. A partir de los resultados de este análisis, los profesionales de Enfermería pueden conocer y unirse a esta u otras comunidades; los investigadores pueden adoptar un análisis similar de las herramientas de comunicación basadas en plataformas digitales; y los responsables de tomar decisiones y otras partes interesadas pueden proponer e incorporar el uso de las TIC en el ámbito de la práctica, la educación y la investigación en Enfermería.

Como limitación, se entiende que los mensajes fueron analizados sin un abordaje complementario para explorar los temas con los participantes; sin embargo, se considera que la participación de enfermeras y enfermeros de diferentes países y la relevancia de los temas explorados contribuyen al análisis y la reflexión de temas relacionados con la educación y la práctica de Enfermería en la Región de las Américas.

## Conclusión

Este estudio tuvo como objetivo analizar el perfil de uso y el contenido de los mensajes de la Comunidad de Práctica de Enfermería en la Región de las Américas de la OPS.

Los participantes de la CdP, procedentes de 20 países, son en su mayoría mujeres, con edades comprendidas entre 26 y 35 años, profesionales de Enfermería, con titulación universitaria y que trabajan en instituciones hospitalarias. Los hallazgos muestran que la plataforma en línea ha permitido el uso de foros de discusión de fácil acceso y con flexibilidad en cuanto a espacio, tiempo e idioma, además de la construcción de redes profesionales y el intercambio de experiencias, materiales, recursos actualizados y oportunidades de desarrollo profesional.

Los temas analizados (educación, empleo, liderazgo y prestación de servicios) se abordaron desde la perspectiva de profesionales de distintos países, presentando los retos a los que se enfrentan y sus reflexiones sobre la situación de la Enfermería en la Región. A través de la Comunidad de Práctica fue posible debatir temas importantes para el contexto de la Enfermería en la Región y aumentar el compromiso, la cooperación y la solidaridad de los profesionales de Enfermería para el aprendizaje colectivo y el desarrollo de capacidades, maximizando las contribuciones de las enfermeras y los enfermeros a los servicios y sistemas de salud.

Así, se espera que más enfermeras, enfermeros y otras partes interesadas puedan unirse a la Comunidad de Práctica y contribuir de forma colaborativa al avance de la Enfermería en la Región de las Américas.

# Translation: Comunidade de Prática de Enfermagem: conectando enfermeiros e enfermeiras na Região das Américas *

**DOI:** 10.1590/1518-8345.7254.4402

**Published:** 2024-11-15

**Authors:** Bruna Moreno Dias, Johel Nazareth Díaz Pérez, Silvia Helena de Bortoli Cassiani

**Affiliations:** ^1^ Organización Panamericana de la Salud, Departamento de Sistemas y Servicios de Salud, Washington, DC, Estados Unidos da América.; ^2^ Organización Panamericana de la Salud, Panamá, Provincia de Panamá, República de Panamá.; ^3^ Organización Panamericana de la Salud, Washington, DC, United States of America.

**Keywords:** Enfermagem, Recursos Humanos de Enfermagem, Tecnologia da Informação, Liderança, Informática em Enfermagem, Fóruns de Discussão

## Abstract

analisar o perfil de utilização e o conteúdo das mensagens da Comunidade de Prática de Enfermagem da Região das Américas da Organização Pan-Americana da Saúde como ferramenta voltada ao compartilhamento de experiências, conhecimentos e oportunidades para enfermeiros e enfermeiras.

estudo descritivo e qualitativo, cujo objetivo foi analisar as mensagens publicadas nos fóruns da Comunidade de Prática. Os dados foram analisados por meio de análise de conteúdo.

a Comunidade de Prática permitiu a participação de 1765 profissionais de 20 países, com 4001 mensagens publicadas. Os temas analisados (educação, emprego, liderança e prestação de serviços) foram abordados na perspectiva de profissionais de diferentes países, apresentando os desafios que enfrentam e os suas reflexões sobre a situação da Enfermagem na Região.

a plataforma permitiu que profissionais de diferentes países e áreas interagissem e debatessem temas relevantes para o ensino e a prática da Enfermagem na Região das Américas. Através da Comunidade de Prática, espera-se aumentar o compromisso, a cooperação e a solidariedade para a aprendizagem coletiva e o desenvolvimento de capacidades, maximizando a contribuição dos enfermeiros com os serviços e sistemas de saúde.

## Destaques

**(1)** A plataforma tem permitido a troca de experiências, conhecimentos e oportunidades.

**(2)** A participação de diferentes atores aumenta o compromisso e a cooperação.

**(3)** É um recurso útil para discutir temas importantes no contexto da Enfermagem.

## Introdução

 Na Região das Américas existem cerca de sete milhões de profissionais de enfermagem, representando 56% da força de trabalho em saúde ^(^ 1 ^)^ . Estes profissionais estão na linha da frente da prestação de serviços e desempenham um papel importante na prestação de cuidados centrados na pessoa e na comunidade. Em muitos países, atuam como líderes ou membros-chave de equipes de saúde multidisciplinares e interprofissionais. Apesar disso, ainda é necessário investir para fortalecer e otimizar esta categoria profissional ^(^ 2 ^)^ . 

Reconhecendo o contexto de mudanças e a complexidade do processo de tomada de decisão em saúde e propondo discussões de alto nível sobre temas relevantes para o contexto da Enfermagem na Região das Américas, desenvolveu-se a Comunidade de Prática de Enfermagem na Região das Américas da Organização Pan-Americana da Saúde (OPAS).

 A Comunidade de Prática (CdP) considera a Diretriz estratégica para a enfermagem na Região das Américas ^(^ 3 ^)^ e a Orientações estratégicas mundiais para enfermeiros e parteiras 2021-2025 da Organização Mundial da Saúde (OMS) ^(^ 4 ^)^ ; e está alinhada à visão compartilhada e colaborativa e à necessidade de investir e fortalecer os recursos humanos para a saúde, especificamente a Enfermagem. 

 A CdP visa reunir enfermeiros e enfermeiras, como assim também outros interessados, potenciar a troca de experiências, informações e conhecimentos e fortalecer a liderança dos enfermeiros em seus espaços profissionais ^(^ 5 ^)^ . Por meio da comunidade, por meio de uma plataforma *online* , espera-se proporcionar um espaço de interação e troca de conhecimentos e experiências entre enfermeiros/as da Região das Américas, além de fortalecer de forma colaborativa os sistemas e serviços para o acesso universal à saúde, a cobertura universal da saúde e o alcance dos Objetivos de Desenvolvimento Sustentável. 

 O conceito de Comunidade de Prática não é novo: foi proposto por primeira vez em 1998 com o propósito de reunir um grupo de pessoas que compartilham interesses comuns, através do diálogo e da reflexão coletiva, para trocar conhecimentos e analisar propostas de soluções práticas para problemas comuns ^(^ 6 ^)^ . 

 Os objetivos de uma CdP podem ser orientados para a resolução de problemas, criação de ideias, distribuição de conhecimento, divulgação de aprendizagens e reflexões coletivas ^(^ 7 ^)^ . 

 Assim, recorrendo às Tecnologias de Informação e Comunicação (TICs) através de fóruns *online* , de fácil acesso e flexíveis em termos de espaço e tempo, é possível: construir redes de apoio ( *networking* e associações); aprender com outras experiências e propor soluções práticas; analisar criticamente a prática e maximizar as respostas; compartilhar materiais e recursos atualizados; e compartilhar oportunidades de desenvolvimento profissional, como eventos e possibilidades de treinamento. 

 Foram identificadas experiências de utilização de comunidades de prática entre professores ^(^ 8 ^-^ 9 ^)^ , pesquisadores ^(^ 10 ^)^ , profissionais de saúde ^(^ 11 ^-^ 12 ^)^ e profissionais de enfermagem ^(^ 13 ^)^ , mas nenhuma com o alcance e a proposta da CdP apresentada neste estudo. 

Diante do exposto, o presente estudo tem como objetivo analisar o perfil de utilização e o conteúdo das mensagens da Comunidade de Prática de Enfermagem da Região das Américas da Organização Pan-Americana da Saúde como ferramenta destinada ao compartilhamento de experiências, conhecimentos e oportunidades para enfermeiros(as).

## Método

### Tipo de estudo

 Estudo qualitativo e descritivo, realizado segundo as diretrizes do *Consolidated Criteria for Reporting Qualitative Research* (COREQ) ^(^ 14 ^)^ , que teve como objetivo analisar o perfil de utilização e conteúdo das mensagens da Comunidade de Prática de Enfermagem na Região das Américas da Organização Pan-Americana de a Saúde, criada em agosto de 2022. 

### Cenário

 A Comunidade de Prática foi desenvolvida com o apoio técnico de um especialista no desenvolvimento de plataformas digitais de aprendizagem. A comunidade está organizada em uma página pública ( https://comunidadenfermeria.paho.org/ ), com informações sobre a iniciativa, espaço para divulgação de eventos, publicações e recursos adicionais e conexão com a Comunidade Mundial de Prática de Enfermagem e Obstetrícia da OMS; além dos fóruns, disponíveis aos participantes cadastrados na plataforma Campus Virtual de Saúde Pública (CVSP/OPAS). Portanto, para acessar a plataforma, o participante deve se cadastrar como usuário no CVSP, sem incorrer em nenhum custo ou necessitar de aprovação prévia. 

A plataforma oferece fóruns abertos para apresentação e interação dos membros sobre temas de interesse da Enfermagem (educação, emprego, liderança e prestação de serviços), acessíveis a todos os participantes, bem como fóruns de acesso restrito, sujeitos à aprovação do moderador de grupos específicos: Projetos da OPAS, Chefes de Enfermagem e Grupo de Profissionais de Enfermagem da América Central e do Caribe (GPECC).

Os fóruns são espaços que permitem a interação entre os participantes, compartilhando ideias, percepções, reflexões, experiências, conhecimentos, dúvidas e oportunidades. Além de mensagens, é possível compartilhar documentos técnicos, artigos, eventos e outras oportunidades. Em cada fórum, os participantes podem propor uma nova discussão ou responder a perguntas e comentários previamente feitos por outros participantes.

 Na CdP também são realizadas atividades de formação, espaço que permite participar em atividades como questionários, webin *ário* s e reflexões. A estrutura e os recursos da Comunidade de Prática são apresentados na Figura 1 . 

**Figura 1 -:** Guia de navegação da Comunidade de Prática. Washington, DC, Estados Unidos da América, 2023

### Período e coleta de dados

Foram analisadas todas as mensagens publicadas entre agosto de 2022 e setembro de 2023 nos fóruns abertos da plataforma (educação, emprego, liderança e prestação de serviços) realizadas pelos usuários, considerados participantes deste estudo. As mensagens foram extraídas da plataforma CdP por meio da ferramenta de emissão de relatório de participação e organizadas em editor de texto para posterior análise.

 Ressalta-se que o relatório de participação foi extraído pela pesquisadora principal, que possui perfil de gestora da plataforma, utilizando ferramenta própria da plataforma para exportar as mensagens do fórum para um arquivo .csv. Não houve contato entre os pesquisadores e os participantes, não havendo possibilidade de influência ou interferência no conteúdo por parte dos pesquisadores. Como as mensagens dos participantes foram extraídas na íntegra, a garantia de confiabilidade não depende de *feedback* ou validação dos participantes. 

Para analisar o perfil de utilização da comunidade, considerou-se o número de participantes, país de origem, sexo, idade, profissão, nível de escolaridade e local de trabalho.

### Processamento e análise de dados

 Os registros de mensagens foram anonimizados e os participantes foram identificados com um código alfanumérico (P1, P2, P3, ...). Os dados foram analisados por meio de análise de conteúdo, com análise lexical e semântica de conteúdo ^(^ 15 ^)^ . As categorias de análise foram identificadas e definidas previamente (categorias *a priori* ), adotando-se os seguintes codificadores: educação, emprego, liderança e prestação de serviços, pois são os quatro eixos estratégicos das Orientações estratégicas mundiais para enfermeiros e parteiras 2021-2025 da OMS ^(^ 4 ^)^ . Não foram criadas subcategorias. 

 Os dados foram analisados por uma pesquisadora e validados por outra. Para o gerenciamento dos dados foi utilizado o *software* Nvivo, versão 14, que dá suporte ao processo analítico e permite organizar os dados e realizar a análise lexical, semântica e de ocorrência/frequência de termos. 

### Aspectos éticos

 O estudo segue diretrizes éticas internacionais para pesquisas relacionadas à saúde em seres humanos ^(^ 16 ^)^ . Devido à utilização de dados de acesso público, o estudo está dispensado de submissão ao Comitê de Ética. Contudo, foi preservada a privacidade e o anonimato dos participantes. Vale salientar que a comunidade distribui conteúdos às vezes fornecidos por terceiros e/ou por seus usuários; portanto, as opiniões, recomendações, declarações e conteúdos expressos ou disponibilizados na Comunidade de Prática são de responsabilidade dos autores e não refletem necessariamente o posicionamento da OPAS. 

## Resultados

### Perfil de uso

 A Comunidade de Prática é composta por 1765 participantes de 20 países das Américas. A maioria dos participantes são do Equador (41,8%) e Peru (30,3%), mulheres (87,8%), com idade entre 26 e 35 anos (29,9%), profissionais de enfermagem (87,1%), com formação universitária (57,9%) e trabalham em instituições hospitalares (30,5%), conforme Tabela 1 . 

**Tabela 1 -:** Caracterização dos participantes da Comunidade de Prática (n = 1765). Washington, DC, Estados Unidos da América, 2023

**Variáveis**	**n**	**%**
**País**		
Equador	737	41,8
Peru	534	30.3
Colômbia	427	24.2
México	13	0,7
Bolívia	13	0,7
Argentina	9	0,5
Uruguai	5	0,3
Nicarágua	4	0,2
República Dominicana	4	0,2
Panamá	2	0,1
Paraguai	2	0,1
Chile	2	0,1
Guatemala	2	0,1
Brasil	1	0,1
El Salvador	1	0,1
Cuba	1	0,1
Domínica	1	0,1
Venezuela	1	0,1
Estados Unidos	1	0,1
Honduras	1	0,1
Outros	4	0,2
**Gênero**		
Feminino	1549	87,8
Masculino	214	12.1
Não informado	2	0,1
**Idade (anos)**		
[26-35]	528	29,9
[36-45]	416	23,6
[18-25]	396	22.4
[46-55]	275	15.6
>56	143	8.1
Não informado	7	0,4
**Local de trabalho**		
Hospital	538	30,5
Centro de Saúde	470	26,6
Gestão e Administração de Serviços de Saúde	203	11,5
Não informado	554	31.4
**Educação**		
Nível Universitário	1022	57,9
Mestrado	322	18.2
Estudante de Graduação	133	7,5
Doutorado	42	2.4
Técnico	24	1.4
Outro	206	11.7
Não informado	16	0,9
**Profissão**		
Profissionais de Enfermagem	1537	87,1
Profissionais de nível médio de Enfermagem	85	4.8
Clínicos Gerais	8	0,5
Psicólogos	5	0,3
Agentes Comunitários de Saúde	4	0,2
Estagiários e Assistentes Médicos	3	0,2
Farmacêuticos	3	0,2
Trabalhadores de cuidados pessoais em instituições	3	0,2
Profissionais de Medicina Tradicional e Alternativa	2	0,1
Dietistas e Nutricionistas	2	0,1
Profissionais de Saúde e Higiene Ocupacional e Ambiental	1	0,1
Médicos especialistas	1	0,1
Profissionais de Serviço Social	1	0,1
Estagiários Paramédicos	1	0,1
Outros	104	5.9
Não informado	5	0,3

No período analisado, foram publicadas 4001 mensagens na CdP, das quais 524 foram realizadas nos fóruns em análise, a saber: educação (n=163), emprego (n=104), liderança (n=124) e prestação de serviços (n=133), sendo que cada fórum contém as mensagens e opiniões na interação dos participantes em um contínuo de comunicação.

As mensagens em análise foram realizadas por 176 participantes de diferentes localizações geográficas, em diferentes dias e horários e majoritariamente em espanhol. Também foram utilizados fóruns para compartilhar artigos e outros materiais.

 As mensagens tiveram extensão média de 81,4 palavras, e entre os termos mais frequentes destacam-se: formação, profissionais, trabalho, liderança, oportunidades, qualidade e fortalecer, como pode ser visto na nuvem de palavras da Figura 2 , que representa visualmente os termos mais frequentemente utilizados nas 524 mensagens publicadas nos fóruns da CdP em análise. 

**Figura 2 -:** Nuvem de palavras dos fóruns da Comunidade de Prática. Washington, DC, Estados Unidos da América, 2023

A análise de conteúdo temático é apresentada a seguir.

### Educação

No que diz respeito à educação, os participantes destacaram a importância da formação do enfermeiro ajustada às necessidades de saúde e de um modelo de cuidados centrado na promoção de saúde e prevenção de doenças. Também consideram oportuno alterar o atual paradigma de ensino, centrando-se nos determinantes sociais da saúde e na atenção primária.

*É preciso que os programas de Enfermagem oferecidos opções ajustadas à realidade social do país* (P6). 

*A formação em Enfermagem exige compreensão dos determinantes socioeconômicos e culturais dos processos saúde-doença, implica o estudo constante da relação entre educação e trabalho, para que a prática pedagógica promova a nova forma de pensar* (P54). 

É enfatizada a necessidade de investir na educação de forma permanente, com oportunidades de formação nos diferentes níveis de cuidados de saúde e ao longo da vida profissional.

*É preciso também dar continuidade à educação em serviço, fortalecendo a prática dos enfermeiros que estão nos serviços dos diferentes níveis de atenção* (P18). 

Para tanto, embora a incorporação de tecnologias de informação e comunicação seja considerada um desafio, entende-se que esses recursos são essenciais para ampliar o alcance das ações e estratégias de formação e desenvolvimento profissional por meio da virtualidade.

*É importante que o enfermeiro tenha oportunidade de formação virtual, pois o horário em que estamos trabalhando não permite sair e treinar presencialmente* (P78). 

*Um dos desafios e oportunidades são as tecnologias de informação e comunicação* (P120). 

Além disso, é necessário aumentar a oferta de vagas e o acesso aos cursos de pós-graduação, com maior investimento em pesquisas, desenvolvendo novos conhecimentos e competências, bem como possibilitar a formação de professores e ampliar a capacidade de formação de novos recursos humanos em Enfermagem.

*Um dos desafios é a formação acadêmica, principalmente no que diz respeito à obtenção de um diploma de nível superior em Enfermagem como os mestrados; são poucos e seu acesso também é limitado* (P156). 

*Falta muita coisa na área de ensino de novas técnicas ou avanços que incentivem a pós-graduação ou o doutorado, e que possam ser realizados dentro do país* (P150). 

### Emprego

Quanto ao emprego, reforça-se a intersecção dos sectores econômico e social, apontando a necessidade de uma posição política perante os diferentes atores e líderes políticos.

*Além disso, a questão do emprego é altamente complexa e depende do contexto político e social de cada país, onde existe uma diversidade de interesses entre aqueles que participam na tomada de decisões* (P54). 

O gênero foi um dos pontos que mais repercutiu entre os participantes, especialmente a disparidade salarial entre mulheres e homens em cargos semelhantes e a necessidade de mais mulheres em cargos de liderança.

*É muito claro que abordar as questões por detrás das disparidades salariais tem um impacto que vai além do aumento do salário das mulheres: é uma das formas mais importantes de combater a discriminação baseada no sexo no local de trabalho e de promover a igualdade de gênero* (P55). 

Além do gênero, também foram constatadas outras formas de discriminação baseadas em situações de vulnerabilidade.

*As desigualdades de gênero continuam sendo um tema de discussão e, embora persistam, deve-se reconhecer que em alguns países está sendo feito trabalho sobre a questão para minimizar os riscos, especialmente em grupos mais vulneráveis, como mulheres indígenas, afrodescendentes, lésbicas, gays e bissexuais, transgênero e intersexuais (LGBTI) e migrantes, por isso acredito que devemos trabalhar duro em estratégias educacionais para mudança em comunidades e instituições com pensamento crítico para culturalizar e melhorar a dinâmica social* (P22). 

Também foi abordada a problemática salarial da profissão, com debates sobre a incompatibilidade entre salários e níveis de formação e experiência, a recomendação de estabelecimento de pisos salariais e as repercussões negativas dos baixos salários.

*Ter um salário que corresponda ao nível de formação, mas muitas vezes isso não é cumprido e os profissionais que investem tempo e dinheiro em cursos de pós-graduação o fazem mais pela satisfação pessoal do que para obter algum tipo de reconhecimento* (P80). 

Foram destacadas as diferenças salariais entre os setores público e privado, bem como nos diferentes modelos de contratação, com flexibilidade laboral, subemprego, falta de garantias de emprego e pouca estabilidade no emprego. Este debate foi relacionado à temática das condições de trabalho e do trabalho digno.

*Há uma certa disparidade nos salários e na forma de contratação, inclusive são oferecidos salários inferiores ou iguais aos de um Auxiliar de Enfermagem* (P6). 

*O trabalho da enfermagem deve obedecer às características do Trabalho Decente, o que implica acesso a: salários justos, segurança social, formação profissional, igualdade de género, livre de riscos e direitos laborais em igualdade de condições para todos* (P133). 

### Liderança

Em relação à liderança, os participantes reconhecem a sua importância para o alcance dos objetivos e melhor desempenho das suas atividades, mesmo que isso represente um desafio.

*A prática profissional traz consigo novos desafios todos os dias, sobretudo na consolidação do que foi aprendido e na formação, atualização e preparação para responder às necessidades que surgem* (P58). 

Dado o papel que os enfermeiros desempenham nos serviços de saúde, destaca-se a necessidade de que tenham mais espaço para a liderança, especialmente nos serviços de nível primário.

*Não há espaços para líderes de enfermagem no primeiro nível de atenção à saúde, a título de verba, então eles assumem esse papel, fazem isso pela profissão, mas sem reconhecimento econômico ou administrativo, e isso, ao invés de motivar pode desmotivar, já que liderar uma equipe implica muito mais responsabilidades* (P147). 

*Também não há espaço para líderes de enfermagem no primeiro nível de atenção à saúde, reconhecemos o trabalho que a enfermeira realiza nesse nível de atenção; é a espinha dorsal de um estabelecimento de saúde, liderando a equipe de saúde no cuidado ao indivíduo, à família e à comunidade com liderança, dedicação, perseverança em áreas remotas e às vezes até geograficamente inacessíveis* (P21). 

Para isso, é necessário melhorar a formação do pessoal de enfermagem, em termos de conhecimentos e competências, com oportunidades formais de desenvolvimento de liderança.

*Programas de liderança devem ser estabelecidos ou organizados para incentivar o desenvolvimento de líderes entre os jovens profissionais de Enfermagem* (P18). 

Isso inclui a adoção de ferramentas de gestão, o reforço do pensamento crítico e o aumento da capacidade de gerar, analisar e utilizar dados para a tomada de decisão.

*A formação em liderança deve começar nas aulas; o pensamento crítico, a pesquisa e a inovação devem ser fortalecidos; devemos incentivar a ampliação do nosso conhecimento* (P63). 

Por fim, na perspectiva do desempenho da liderança, foi destacada a ampliação do papel dos enfermeiros e enfermeiras, através da implementação do papel do Enfermeiro de Práticas Avançadas, com atuação autônoma e independente.

*Acredito que fortalecer a prática avançada dos profissionais de Enfermagem que estão no dia a dia com os sujeitos e as populações que necessitam de cuidado contribuiria para melhorar essa capacidade de liderança na sua área de atuação prática* (P177). 

### Prestação de serviços

No que diz respeito à prestação de serviços, destacou-se a capacidade resolutiva dos sistemas de saúde, especialmente do primeiro nível de atenção em rede com outros serviços de saúde, para que os profissionais de enfermagem possam atuar de forma adequada para atender as necessidades de saúde da população.

*A capacidade de resposta dos sistemas de saúde envolve enfrentar diversos desafios, como mudanças socioeconómicas e políticas, modificações no perfil epidemiológico, surtos de doenças e alterações climáticas* (P3). 

*Relativamente à questão da referência e contrarreferência, existem limitações no primeiro nível de cuidados de saúde devido à infraestrutura inadequada e à limitada capacidade de resolução de alguns Estabelecimentos de Saúde* (P21). 

A escassez de recursos humanos para a saúde, especialmente de profissionais de Enfermagem, foi destacada como fator limitante para a prestação de serviços de saúde, o que gera impactos negativos nas condições de trabalho e na saúde mental dos profissionais. Da mesma forma, foram abordadas dificuldades relacionadas à estrutura de atendimento, com recursos financeiros e materiais limitados, e impacto negativo na segurança do trabalho.

*A falta de recursos humanos nos impede prestar cuidados de melhor qualidade, produzindo uma sobrecarga de trabalho, razão pela qual a Saúde Mental é afetada, há défice de sistemas informáticos, falta de equipamentos e mobiliário e infraestruturas inadequadas* (P448). 

*A verba destinada à saúde deve ser aumentada porque, devido à falta de insumos nas unidades de saúde, é difícil prestar cuidados de qualidade* (P135). 

Por fim, abordou-se a regulação, destacando a necessidade de revisão dos marcos regulatórios para atualizar aspectos como âmbito de atuação, carga horária e remuneração, além da garantia de direitos dos trabalhadores de Enfermagem.

*Principalmente padronizar horários de trabalho, cargas horárias e funções, para que o trabalho da Enfermagem seja reconhecido como um centro de muitos profissionais* (P61). 

*Políticas, normas, legislação, e, mais do que tudo, uma regulamentação no cumprimento em relação à carga horária e ao incentivo que cada profissional recebe, o reconhecimento da profissão e mudanças de paradigma do sindicato da saúde em relação à atividade profissional dos trabalhadores na área* (P47). 

## Discussão

Os resultados desta análise reforçam a utilidade da Comunidade de Prática como plataforma de intercâmbio de informações e opiniões relevantes para o contexto da Enfermagem na Região das Américas.

 Os fóruns têm permitido a interação de diferentes profissionais, considerando seus contextos locais, expressando-se sob diferentes perspectivas, expressando suas opiniões e compartilhando experiências e sugestões. Alinhados com as orientações estratégicas da OPAS ^(^ 3 ^)^ e da OMS ^(^ 4 ^)^ , os temas concentram-se em áreas que requerem mais investimentos para o avanço da Enfermagem na Região das Américas. 

 A CdP proporciona um espaço seguro e acolhedor para interações, incentivando a participação e a inclusão, promovendo a inovação e a adaptabilidade e reduzindo o isolamento profissional, à medida que os membros desenvolvem um sentido de conexão, confiança mútua, reciprocidade e valorização de opiniões diferentes, tornando-se em uma importante ferramenta para profissionais de saúde ^(^ 17 ^-^ 18 ^)^ . 

 Dos temas discutidos, no que diz respeito à educação, é importante destacar que a Região possui uma formação heterogênea de profissionais de Enfermagem, e que são necessárias mudanças no paradigma educacional que considerem o modelo de atenção centrado nas necessidades de saúde da população e enfatizem o fortalecimento sistemas de saúde com foco na atenção primária ^(^ 19 ^)^ . 

 Outra questão relacionada com a educação foi o investimento na formação de professores, um tema relevante, pois foi observado que alguns professores de países da Região não receberam qualquer capacitação pedagógica para o seu trabalho ^(^ 20 ^)^ . 

 Desta forma, investir em cursos de pós-graduação é uma estratégia para ampliar a capacidade de formação dos enfermeiros(as), sendo uma das ações para enfrentar a escassez de profissionais ^(^ 21 ^)^ . Além disso, os enfermeiros e enfermeiras com doutorado contribuem para melhorar o atendimento ao paciente e desenvolver políticas de saúde ^(^ 22 ^)^ . 

 Investir nesse nível de formação também terá impacto no desenvolvimento de pesquisas, especialmente relacionadas com temas que contribuam para a saúde universal, formando enfermeiros e enfermeiras capacitados para a análise situacional e o uso da melhor evidência para propor soluções aos problemas de saúde que se apresentem ^(^ 23 ^)^ . 

 Neste contexto, resolver a lacuna na oferta de formação de doutorado é um desafio para a Região, uma vez que muitos países da América Latina e do Caribe não oferecem programas de doutorado e 65% desses programas estão no Brasil ^(^ 2 ^)^ . 

 A formação de doutorado e a formação docente podem beneficiar-se da Comunidade de Prática ^(^ 24 ^)^ , como espaço informal de desenvolvimento profissional contínuo, aumentando a capacidade destes profissionais ^(^ 17 ^)^ , bem como o potencial de parcerias entre pesquisadores e profissionais como forma de produzir conhecimentos relevantes para a prática ^(^ 6 ^)^ . 

 Nesse contexto, além de discutir problemas e soluções para a formação em Enfermagem, a Comunidade de Prática se destaca como um espaço de aprendizagem dinâmico e colaborativo no qual são compartilhados conhecimentos e oportunidades de aprendizagem ^(^ 13 ^)^ . 

 No que diz respeito à liderança, entende-se que os enfermeiros devem ser considerados no desenvolvimento e aplicação das políticas governamentais e que devem estar presentes nos cargos de decisão nas organizações do sistema de saúde e nos departamentos governamentais. Dessa forma, são essenciais estratégias formais para estimular o crescimento e o desenvolvimento dos novos profissionais de Enfermagem ^(^ 2 ^)^ . Neste contexto, sabe-se que as interações que ocorrem na CdP são úteis para a prática profissional e para reforçar a identidade e o papel profissional ^(^ 25 ^)^ . 

 É importante destacar que, os temas discutidos nos fóruns estão alinhados com a “Política sobre a força de trabalho em saúde para 2030: fortalecendo os recursos humanos em saúde para alcançar sistemas de saúde resilientes”, aprovada pelo 60º Conselho Diretor da OPAS em setembro de 2023, que propõe ações prioritárias: a) Reforçar a governança e promover políticas e planos nacionais de recursos humanos em saúde; b) Desenvolver e consolidar mecanismos regulatórios relacionados com os recursos humanos em saúde; c) Fortalecer a formação e a implementação de equipes interprofissionais em redes integradas de serviços de saúde baseadas na atenção primária à saúde; d) Melhorar o desenvolvimento e reforçar as capacidades do pessoal de saúde para abordar as prioridades das populações e para a preparação e resposta a emergências de saúde pública; e e) Promover condições de trabalho dignas e a proteção física e mental dos trabalhadores da saúde, além de uma provisão adequada de recursos humanos para a saúde através de financiamento e regulamentação ^(^ 26 ^)^ . Assim, a política apresenta elementos e contextualiza as questões discutidas na CdP, servindo de subsídio para que tomadores de decisão e demais atores de diversos setores proponham ações estratégicas voltadas às mudanças necessárias nos sistemas de saúde, a fim de fortalecer os recursos humanos para a saúde. 

Dessa forma, entende-se que os participantes da CdP demonstram consciência dos problemas práticos dos recursos humanos para a saúde e a enfermagem, trocam opiniões e buscam soluções para esses problemas. A relevância e complexidade dos temas discutidos reforçam a importância da CdP como espaço de interação, com forte relação dialógica.

Apesar dos resultados positivos obtidos em um ano de aplicação da CdP e seus potenciais usos, entende-se que o comprometimento e a sustentabilidade dos debates e o uso das TICs se consolidam como limitações.

 Envolver os participantes e manter as discussões é um desafio. Entende-se que, a CdP é “fornecedora de respostas” e, para isso, é necessário que haja questionamentos. Consequentemente, é importante incentivar a continuidade dos debates, alimentando o grupo com reflexões, questionamentos e esclarecimentos e valorizando seus comentários ^(^ 8 ^)^ . Por outro lado, existem ainda barreiras comuns à utilização de tecnologias digitais, incluindo infraestruturas, tempo, competências, formação, apoio e familiaridade com os recursos digitais ^(^ 27 ^)^ . Isto requer ações que visem a literacia digital e o desenvolvimento de competências em TICs. Apesar destes desafios, entende-se que a CdP constitui uma ferramenta importante para os profissionais da Região. Assim, espera-se ampliar o alcance e a participação de mais enfermeiros. 

A contribuição deste estudo é analisar e divulgar o perfil de utilização e os conteúdos discutidos em uma Comunidade de Prática de Enfermagem. Com base nos resultados desta análise, os profissionais de Enfermagem podem conhecer e integrar esta ou outras comunidades. Os investigadores podem adotar uma análise semelhante das ferramentas de comunicação baseadas em plataformas digitais; e os decisores e outras partes interessadas podem propor e incorporar a utilização das TICs no campo da prática, educação e investigação em enfermagem.

Como limitação, entende-se que as mensagens foram analisadas sem uma abordagem complementar para explorar os temas com os participantes. Contudo, considera-se que a participação de enfermeiros de diferentes países e a relevância dos temas explorados contribuem para a análise e reflexão de temas relacionados à educação e prática de enfermagem na Região das Américas.

## Conclusão

Este estudo teve como objetivo analisar o perfil de utilização e o conteúdo das mensagens da Comunidade de Prática de Enfermagem na Região das Américas da OPAS.

 Os participantes da CdP, provenientes de 20 países, são majoritariamente mulheres, com idades compreendidas entre os 26 e os 35 anos, profissionais de enfermagem, com formação universitária e que trabalham em instituições hospitalares. Os resultados mostram que a plataforma *online* permite a utilização de fóruns de discussão de fácil acesso e flexibilidade de espaço, tempo e idioma, além da construção de redes profissionais e do intercâmbio de experiências, materiais, recursos atualizados e oportunidades de desenvolvimento profissional. 

Os temas analisados (educação, emprego, liderança e prestação de serviços) foram abordados na perspectiva de profissionais de diferentes países, apresentando os desafios que enfrentam e suas reflexões sobre a situação da Enfermagem na Região. Através da Comunidade de Prática, foi possível debater temas importantes para o contexto da Enfermagem na Região e aumentar o compromisso, a cooperação e a solidariedade dos profissionais de Enfermagem para a aprendizagem coletiva e o desenvolvimento de capacidades, maximizando as contribuições dos enfermeiros para os serviços e sistemas de saúde.

Assim, espera-se que mais enfermeiros(as) e outros interessados possam aderir à Comunidade de Prática e contribuir de forma colaborativa para o avanço da Enfermagem na Região das Américas.
